# MicroRNA profiling of the whitefly *Bemisia tabaci* Middle East-Aisa Minor I following the acquisition of *Tomato yellow leaf curl China virus*

**DOI:** 10.1186/s12985-016-0469-7

**Published:** 2016-02-02

**Authors:** Bi Wang, Lanlan Wang, Fangyuan Chen, Xiuling Yang, Ming Ding, Zhongkai Zhang, Shu-Sheng Liu, Xiao-Wei Wang, Xueping Zhou

**Affiliations:** State Key Laboratory of Rice Biology, Institute of Biotechnology, Zhejiang University, Hangzhou, 310058 People’s Republic of China; State Key Laboratory for Biology of Plant Diseases and Insect Pests, Institute of Plant Protection, Chinese Academy of Agricultural Sciences, Beijing, 100193 People’s Republic of China; Ministry of Agriculture Key Laboratory of Agricultural Entomology, Institute of Insect Sciences, Zhejiang University, Hangzhou, 310058 People’s Republic of China; Institute of Biotechnology and Genetic Resources, Yunnan Academy of Agricultural Sciences, Kunming, Yunnan, 650223 People’s Republic of China

**Keywords:** *Tomato yellow leaf curl China virus*, Whitefly *Bemisia tabaci*, Gene silencing machinery, Differentially regulated miRNA profiling

## Abstract

**Background:**

The begomoviruses are the largest and most economically important group of plant viruses exclusively vectored by whitefly (*Bemisia tabaci*) in a circulative, persistent manner. During this process, begomoviruses and whitefly vectors have developed close relationships and complex interactions. However, the molecular mechanisms underlying these interactions remain largely unknown, and the microRNA profiles for viruliferous and nonviruliferous whiteflies have not been studied.

**Methods:**

Sequences of *Argonaute 1*(*Ago1*) and *Dicer 1* (*Dcr1*) genes were cloned from *B. tabaci* MEAM1 cDNAs. Subsequently, deep sequencing of small RNA libraries from uninfected and *Tomato yellow leaf curl China virus* (TYLCCNV)-infected whiteflies was performed. The conserved and novel miRNAs were identified using the release of miRBase Version 19.0 and the prediction software miRDeep2, respectively. The sequencing results of selected deregulated and novel miRNAs were further confirmed using quantitative reverse transcription-PCR. Moreover, the previously published *B. tabaci* MEAM1 transcriptome database and the miRNA target prediction algorithm miRanda 3.1 were utilized to predict potential targets for miRNAs. Gene Ontology (GO) analysis was also used to classify the potential enriched functional groups of their putative targets.

**Results:**

Ago1 and Dcr1orthologs with conserved domains were identified from *B. tabaci* MEAM1. BLASTn searches and sequence analysis identified 112 and 136 conserved miRNAs from nonviruliferous and viruliferous whitefly libraries respectively, and a comparison of the conserved miRNAs of viruliferous and nonviruliferous whiteflies revealed 15 up- and 9 down-regulated conserved miRNAs. 7 novel miRNA candidates with secondary pre-miRNA hairpin structures were also identified. Potential targets of conserved and novel miRNAs were predicted using GO analysis, for the targets of up- and down-regulated miRNAs, eight and nine GO terms were significantly enriched.

**Conclusions:**

We identified Ago1 and Dcr1 orthologs from whiteflies, which indicated that miRNA-mediated silencing is present in whiteflies. Our comparative analysis of miRNAs from TYLCCNV viruliferous and nonviruliferous whiteflies revealed the relevance of deregulated miRNAs for the post-transcriptional gene regulation in these whiteflies. The potential targets of all expressed miRNAs were also predicted. These results will help to acquire a better understanding of the molecular mechanism underlying the complex interactions between begomoviruses and whiteflies.

**Electronic supplementary material:**

The online version of this article (doi:10.1186/s12985-016-0469-7) contains supplementary material, which is available to authorized users.

## Background

RNA silencing, including post-transcriptional gene silencing (PTGS) in plants, RNA interference (RNAi) in animals and gene quelling in fungi, represents a sequence-specific RNA degradation mechanism directed against invasive nucleic acid molecules, which plays an evolutionarily conserved role in gene regulation and defense [1–3]. Recently, significant progress has been made in understanding the various silencing pathways. At least three basic silencing pathways have been identified: (1) siRNA-mediated degradation of abundant or aberrant mRNAs (PTGS or RNAi); (2) microRNA (miRNA)-mediated silencing involved in translational inhibition or degradation of mRNAs; and (3) siRNA-directed *de novo* methylation of DNA and histone proteins, leading to transcriptional gene silencing (TGS).

miRNAs are small 19–24 nucleotide (nt) RNAs that play critical roles in diverse biological processes. In the nucleus, the primary transcript (pri-miRNA), from which the miRNA is derived, can be several kilobases in size and generally transcribed by RNA polymerase II [4, 5]. The pri-miRNA is then processed by Dicer-1 (Dcr1 or Dicer-like1) into the precursor miRNA (pre-miRNA), which is further processed into the mature miRNA-miRNA* duplex [6–8]. This duplex is transported into the cytoplasm, unwound and loaded into an Argonaute (Ago) protein, which is part of the RISC (RNA induced silencing complex) and guides RISC to cleave or suppress target mRNA [6, 7, 9]. In animals, it has been shown that miRNAs can repress the expression of target genes by binding to sequences in both the 3′-UTR [10, 11] and the protein-coding region [12, 13].

The whitefly *Bemisia tabaci* causes severe crop losses by direct feeding on plants as well as vectoring more than 200 different species of begomoviruses [14–16]. Recent phylogenetic analyses and crossing experiments have indicated that the whitefly *B. tabaci* is a complex containing at least 34 morphologically indistinguishable species [17–20]. Within this whitefly complex, the Middle East-Asia Minor 1 (MEAM1) [21–23], previously referred to as the “B biotype”, has become an international concern since the 1980s because of its rapid spread [17, 24–26]. With invasions of whiteflies from this complex, diseases caused by begomoviruses simultaneously increase and pandemics have been frequently recorded in tobacco, tomato, pumpkin, papaya and some other crops throughout the world [15, 27–31]. Among them, one of the major causative agents of begomovirus diseases in Southwest China is *Tomato yellow leaf curl China virus* (TYLCCNV) [32].

The RNAi pathway is functional in whiteflies [33–35] and RNAi has been speculated to be responsible for the inhibition of viral gene expression following acquisition of geminiviruses by whiteflies [36]. However, the roles of RNAi in the complex interactions between begomoviruses and the whitefly remain largely unknown, and miRNA profiles for viruliferous and nonviruliferous whiteflies have not been reported. In this study, we first demonstrated that the core miRNA pathway machinery is present in the whitefly *B. tabaci* MEAM1. We then: (1) investigated the expression profiles of miRNAs in viruliferous and nonviruliferous whiteflies, by utilizing deep sequencing; (2) identified the conserved and novel miRNA candidates of whitefly; and (3) identified targets of the differentially regulated miRNAs in viruliferous and nonviruliferous whiteflies. Our objective is to gain a better understanding of the role of miRNA in the complex interactions between the whitefly vector and TYLCCNV.

## Results and discussion

### Identification of Argonaute 1 and Dicer 1 orthologs in whiteflies

**Fig. 1:**
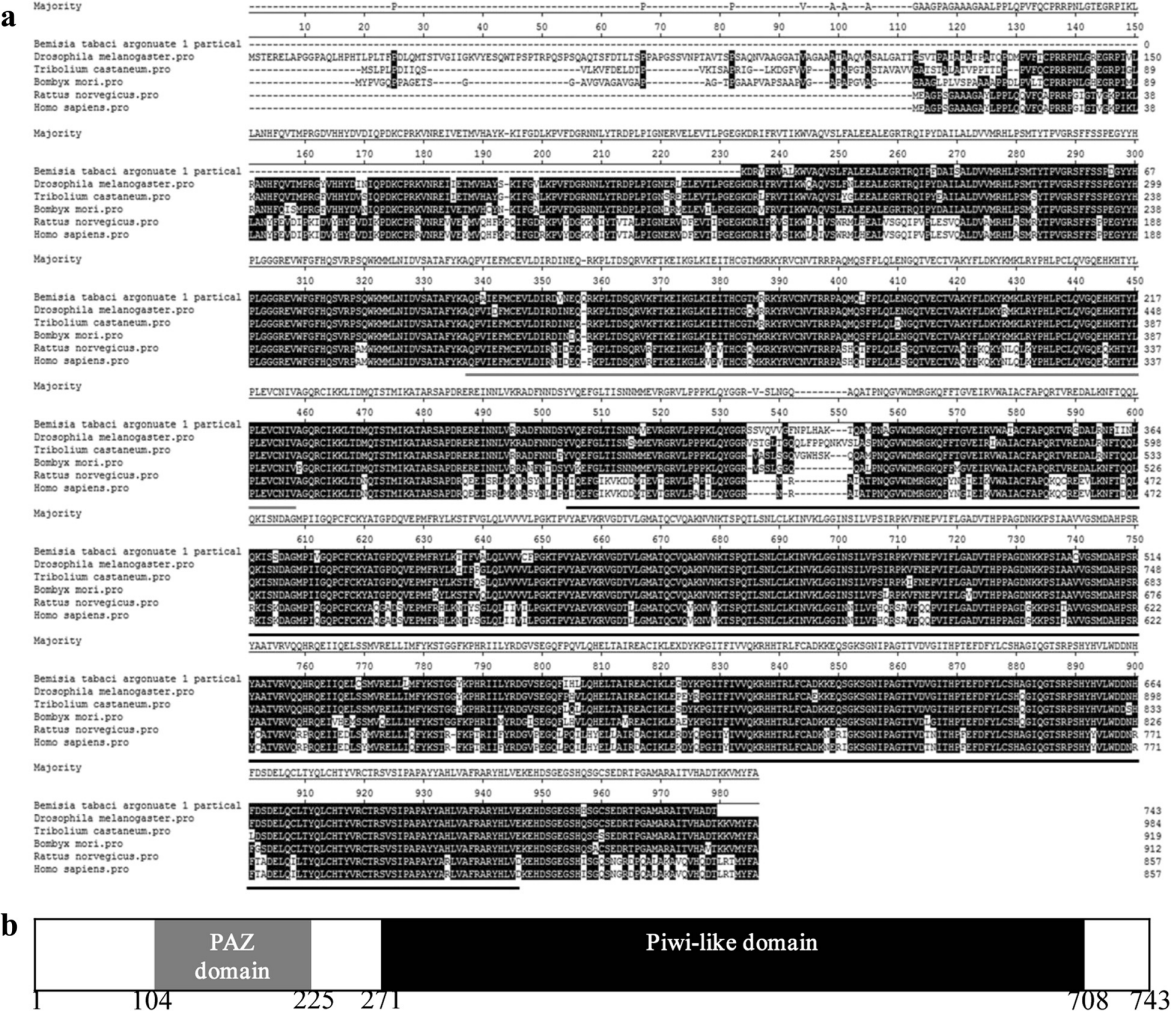
**a** Sequence alignment of Argonaute 1 (Ago1) from whitefly *B. tabaci* MEAM1 and other species including *Drosophila melanogaster, Tribolium castaneum, Bombyx mori, Rattus norvegicus* and *Homo sapiens.* **b** Structure of the partial Argonaute proteins predicted using the NCBI Conserved Domains Server. Conserved PAZ and Piwi-like domains were identified in the assembled *B. tabaci* Ago1

**Fig. 2:**
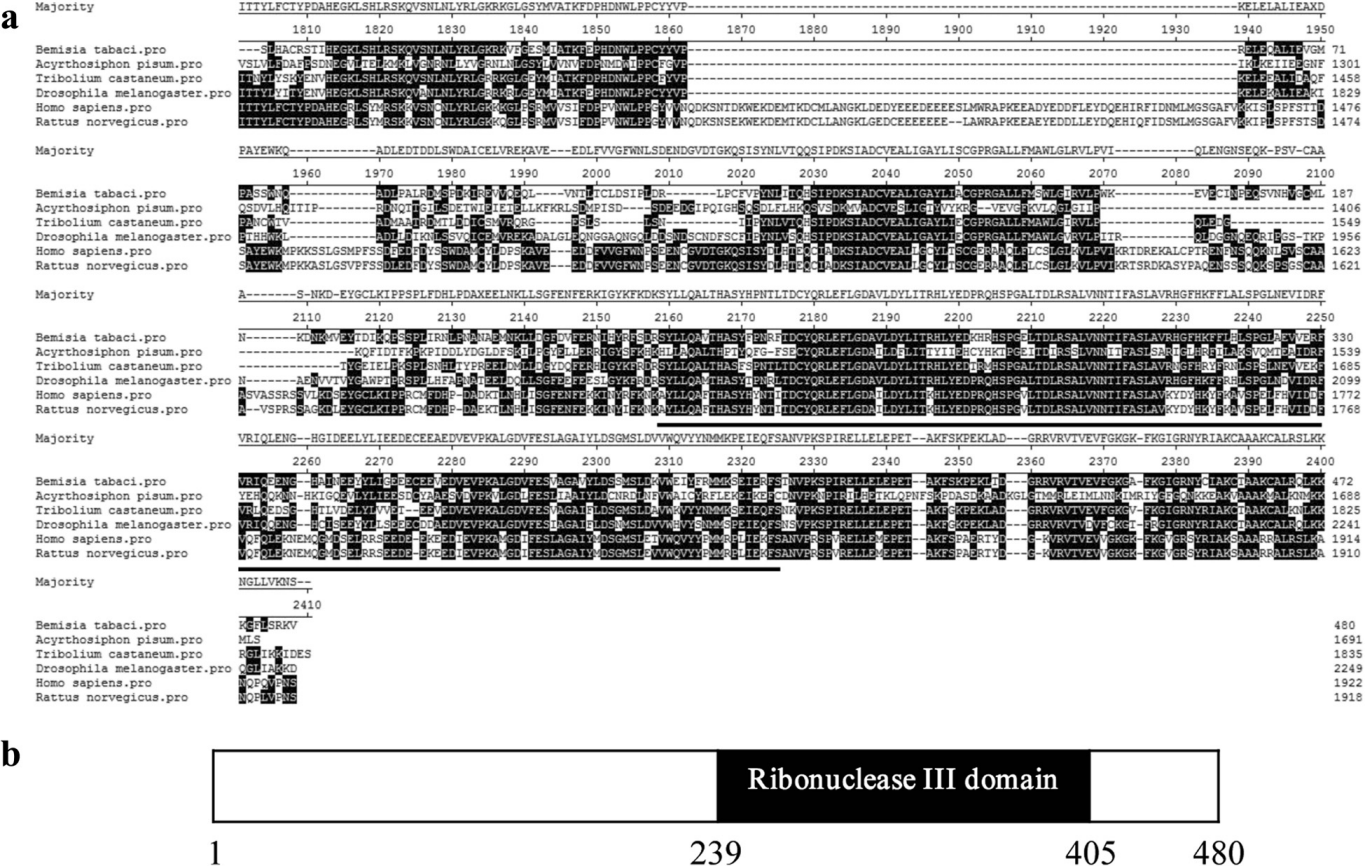
**a** Sequence alignment of Dicer 1 (Dcr1) from whitefly *B. tabaci* MEAM1 and other species including *Drosophila melanogaster, Acyrthosiphon pisum, Tribolium castaneum*, *Rattus norvegicus* and *Homo sapiens.* **b** Structure of partial Dicer proteins predicted using the NCBI Conserved Domains Server. The conserved Ribonulease III domain was identified in the assembled *B. tabaci* MEAM1 Dcr1

*Ago1* and *Dcr1* genes are key elements involved in miRNA-mediated silencing. To determine whether the core RNA-induced gene silencing machinery is present in *B. tabaci* MEAM1, we cloned partial sequences for Ago1 and Dcr1 orthologs. A partial fragment of a putative whitefly *Ago1* gene (encoding 743 amino acids) was sequenced and compared to orthologs from other species, including *Drosophila melanogaster*, *Tribolium castaneum*, *Bombyx mori*, *Rattus norvegicus* and *Homo sapiens*. As expected, all the sequences analyzed were conserved and the typical PAZ and Piwi-like domains reported for other Argonaute family proteins were also present in the whitefly Ago1 (Fig. 1). The entire 743 amino acids of the cloned *B. tabaci* MEAM1 Ago1 exhibited approximately 90 % sequence identity to Ago1 from other insects (Fig. 1a), and approximately 95 and 90 %, within the PAZ and Piwi-like motifs respectively (Fig. 1b). Similarly, the partial sequence of the whitefly *Dcr1* gene (encoding 480 amino acids) was also conserved amongst different insects and the typical Ribonulease III domain reported for other Dicer family proteins was also present in whitefly Dcr1 (Fig. 2). The 480 amino acids of *B. tabaci* MEAM1 Dcr1 exhibited 70 % sequence identity to Dcr1 from other insects (Fig. 2a) and approximately 77 % identity for the Ribonulease III motif (Fig. 2b). These results indicate that two of the core components of the miRNA-mediated silencing system exist in the whitefly.

The presence of this system in whiteflies has implications regarding recent studies demonstrating differences in the global gene expression profile in viruliferous and nonviruliferous whiteflies [37]. It has been found that after TYLCCNV infection a number of genes involved in cell cycle regulation, primary metabolism, the immune response, Toll-like signaling and mitogen-activated protein kinase (MAPK) pathways were differentially regulated in the viruliferous whiteflies [37]. As miRNAs are now recognized as critical regulators of gene expression, we suggest that identification and comparison of miRNAs in viruliferous and nonviruliferous whiteflies could provide new information concerning biological changes in the vector upon virus infection.

### Overview of the analysis of small RNA libraries

**Fig. 3:**
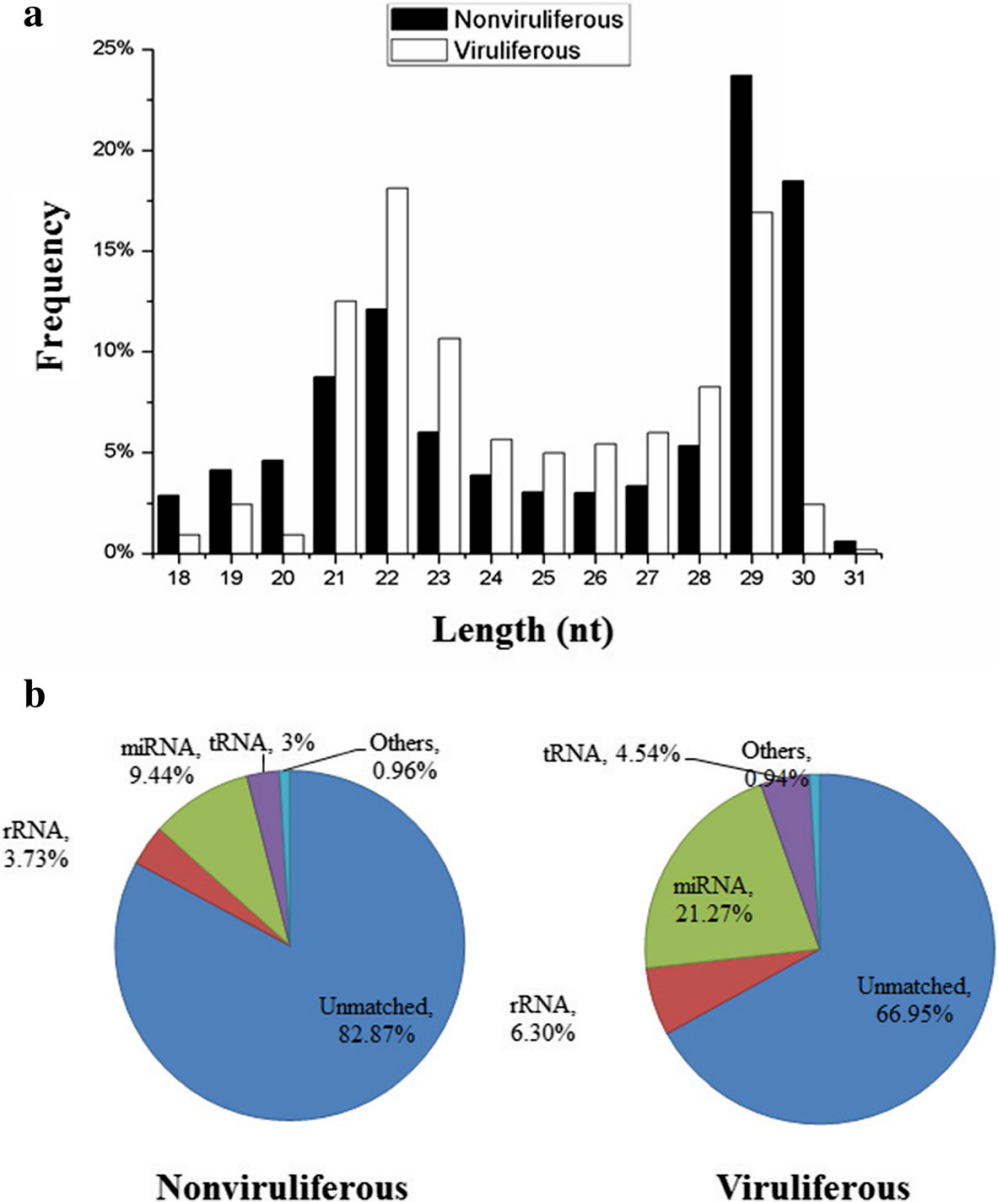
Size distribution (**a**) and coverage (**b**) of small RNAs identified in libraries from nonviruliferous and viruliferous whiteflies

In order to identify differentially expressed miRNAs involved in begomovirus-whitefly interactions, we constructed small RNA libraries from uninfected and TYLCCNV-infected whiteflies. High throughput Solexa sequencing of these two small RNA libraries was performed and low-quality sequences and sequences <18 nt or >31 nt were eliminated. A total of 1,910,274 reads (948,290 unique sequences) in the nonviruliferous library and 5,663,142 reads (1,910,584 unique sequences) in the viruliferous library were obtained. Analysis of the size distribution indicated that the highest percentage of small RNAs in both libraries were 21–23 nt (26.9 and 47.5 % of all reads in nonviruliferous and viruliferous libraries respectively) and 28–30 nt (47.5 and 27.6 % of all reads in nonviruliferous and viruliferous libraries respectively) in length (Fig. 3a). The small RNAs in the 21–23 nt range are consistent with that observed for miRNAs in animals [38], and small RNAs in the 28–30 nt range are consistent with pi-RNA-like sequences. A previous study on identification of miRNAs in *B. tabaci* B and Q has showed that the distribution of sequence lengths from both B and Q libraries were enriched with small RNAs of 21–23 and 28–30 nt [39]. Our data are consistent with this previous report for nonviruliferous whiteflies, which provide confidence in the reliability of the data.

Subsequent sequence analysis (NCBI GenBank and Rfam Version 10.1) indicated that, among these small RNAs, a total of 128,523 (nonviruliferous) and 613,517 (viruliferous) were rRNAs (71,238/3.73 % for nonviruliferous library and 356,499/6.30 % for viruliferous library), snRNAs (13/0.00; 35/0.00 %, respectively), or tRNAs (57,272/3.00; 256,983/4.54 %, respectively) (Fig. 3b). After eliminating reads corresponding to these RNAs, both libraries contained a large fraction of reads derived from unannotated genome sites (82.87 and 66.95 %, respectively) and miRNAs (9.44 and 21.27 %, respectively) (Fig. 3b). These unique data sets and read counts were used to identify conserved and novel miRNAs in whiteflies. The coverage of small RNAs is also consistent with previous report for nonviruliferous whiteflies [39]. It is interesting that the corresponding coverage of reads for rRNAs, snRNAs or tRNAs shows a similar trend in both libraries except for the miRNAs, which were expressed at a much higher level in the library from viruliferous whiteflies.

### Differentially expressed conserved miRNAs in viruliferous relative to nonviruliferous whiteflies

To identify conserved miRNAs in *B. tabaci* MEAM1, all clean small RNA tags were annotated into different categories to remove rRNAs, tRNAs, snRNAs, and snoRNAs using the Rfam database (Version 10.1). The remaining small RNAs from the nonviruliferous and viruliferous whitefly libraries were used to identify conserved miRNAs in *B. tabaci* MEAM1 by comparison to known miRNAs in the miRBase database (Version 19.0). Sequences in our libraries identical to or related to (having four or fewer nucleotide substitutions) miRNA sequences of *D. melanogaster* or other insects (*Aedes aegypti*, *Apis mellifera*, *B. mori*, and *T. castaneum*) were considered to be potentially conserved miRNAs. After BLASTn searches and further sequence analysis, a total of 112 conserved miRNAs were identified from the nonviruliferous whitefly library and 136 conserved miRNAs were identified from the viruliferous whitefly library (Additional file 1: Table S1).

**Fig. 4:**
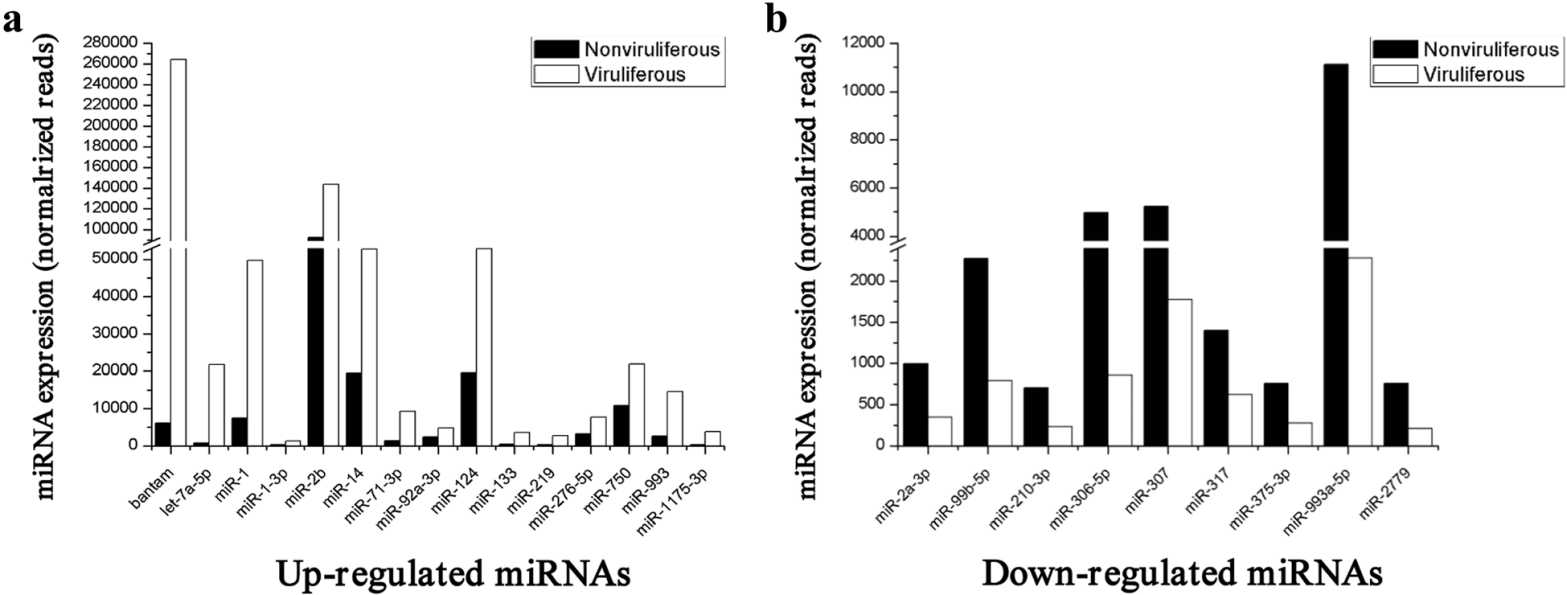
Expression patterns of deregulated conserved miRNAs identified in libraries from nonviruliferous and viruliferous whiteflies. The left panel (**a**) shows up-regulated miRNAs and the right panel (**b**) represents down-regulated miRNAs in viruliferous as compared to nonviruliferous whiteflies. Only miRNAs with at least a two-fold difference in expression levels and an expression of at least 0.1 % in both whitefly libraries are shown

We then compared expression levels of putative conserved miRNA between nonviruliferous and viruliferous whitefly libraries. For the comparison, we first normalized the expression of miRNAs (normalized expression = actual miRNA count/total count of clean reads × 100000), and then set a threshold of a 2-fold difference in normalized expression and a representation of 0.1 % (actual miRNA count/total count of clean reads) in both miRNA libraries. Between the nonviruliferous and viruliferous libraries, we identified 52 miRNAs that were differentially expressed. Among these, 26 miRNAs were unique to the viruliferous library and 2 miRNAs were unique to the nonviruliferous library (Additional file 2: Table S2). Among the 24 miRNAs that were identified in both libraries, 15 miRNAs were up-regulated and 9 reduced relative to the nonviruliferous library (Fig. 4). The strongest relative induction was observed for bantam (43-fold), let-7a-5p (26-fold), miR-1175-3p (13-fold) and miR-219 (10-fold), while the strongest reduction was observed for miR-306-5p (6-fold), miR-993a-5p (5-fold), miR-2779 (4-fold) and miR-307 (3-fold) (Fig. 4).

Bantam miRNA has been reported to simultaneously stimulate cell proliferation and prevent apoptosis in response to patterning cues in *Drosophila* [40]. In our study, bantam miRNA was significantly up-regulated in the viruliferous whiteflies as compared to the nonviruliferous controls. It has been reported that TYLCCNV can activate whitefly immune responses, including autophagy. The induction of autophagy can inhibit cell growth and induce apoptotic cell death, which might lead to a gradual decrease of viral particles within the body of viruliferous whiteflies [37, 41]. An enhanced level of bantam in the viruliferous whiteflies suggests that bantam may act to arrest the apoptotic response and help to maintain homeostasis in the presence of virus.

The let-7 miRNA family, which includes let-7a, let-7b, let-7c, let-7d, let-7e, let-7f, has been known to play an important role in cell cycle, proliferation and apoptosis. Moreover, let-7 has been implicated in post-transcriptional control of responses to pathogenic agents [42, 43]. Significant up-regulation of let-7 was also detected in viruliferous whiteflies, suggesting that this miRNA may act to perturb the cell cycle in the whitefly, thus offering one explanation for the negative effect of this virus on the longevity and fecundity of *B. tabaci* MEAM1. Recently, miR-219 has been connected with NMDA receptor signaling in humans, and it has been shown that deregulation of this miRNA can lead to the development of mental disorders such as schizophrenia [44]. Viruliferous whiteflies also showed a significant increase in miR-219 expression level, although how this is relevant to the presence of virus in the whitefly is unknown. While the physiological functions of other up- and down-regulated miRNAs are still unknown, their specific expression patterns indicate that they are also likely to play critical roles in hypometabolic processes in whiteflies. Further experiments are needed to elucidate their potential roles in this process.

### Identification of novel miRNA candidates

**Fig. 5:**
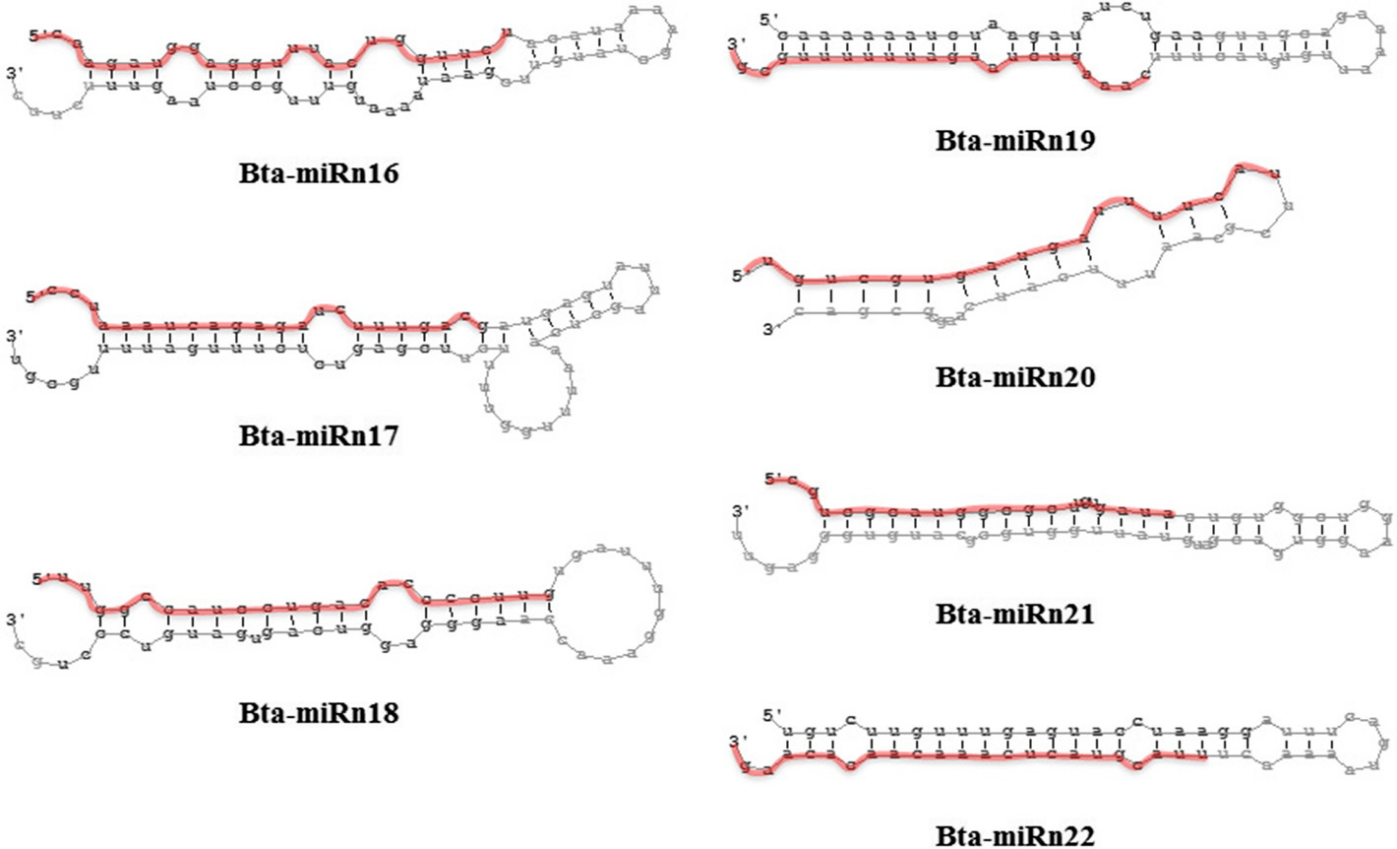
Secondary structures of putative precursor hairpins corresponding to seven novel miRNA candidates. The predicted miRNA mature sequences are highlighted in red

Next, we used the miRNA prediction software miRDeep2 [45] to identify putative novel miRNAs by searching against the previously published *B. tabaci* MEAM1 transcriptome database [46]. In total, 7 potential novel miRNA candidates were identified from both the nonviruliferous and viruliferous libraries (Table 1). The length of the 7 predicted novel miRNA candidates ranged from 18 to 23 nt. The miRDeep2 scores for these novel miRNA candidates were all ≥4. Interestingly, we found that the expression levels of all 7 novel miRNA candidates were up-regulated in the viruliferous as compared to the nonviruliferous library. To investigate whether these novel miRNA candidates are conserved in a wide range of animal species, we used these miRNAs as query sequences to perform BLASTn search against all nucleotide sequences in miRBase Version 19.0. The results indicated that these miRNAs candidates were not found in other species, possibly indicating that they are specific to whiteflies and thus have a species-specific role(s). Interestingly, we found the sequence of bta-miRn16 also map into the genome of a primary endosymbiont, *Candidatus* Portiera aleyrodidarum [47], which has been found to accompany whiteflies in our laboratory without any treatment [48, 49]. Furthermore, to evaluate the novel miRNA candidates represent true miRNAs, the hairpin structures for putative pre-miRNA hairpins were generated using RNAfold [50]. As shown in Fig. 5, although differing complexities, all novel miRNA candidate precursor sequences fold into hairpin structures characteristic of miRNA precursors.

**Table 1:** Novel miRNA candidates identified from whitefly *B. tabaci* MEAM1

Name	Sequence (5′-3′)	Length (nt)	miRDeep2 score	Reads in libraries	
				Nonviruliferous	Viruliferous
bta-miRn16	caagauggagguuuacugguucu	23	971.7	140	1114
bta-miRn17	ccuaaaucagagaucuuugacg	22	213.1	81	121
bta-miRn18	uuggccauccugacaccccuug	21	99.6	13	102
bta-miRn19	caaagucuaagauuuuuugcg	21	21.2	5	20
bta-miRn20	ugucgugaugauuuucau	18	4.2	4	8
bta-miRn21	cgucgcauggcgcuugugaua	21	4.1	1	4
bta-miRn22	uuacguacucaaacaacacaag	22	4	35	143

### Validation of miRNA sequencing data by qRT-PCR

**Fig. 6:**
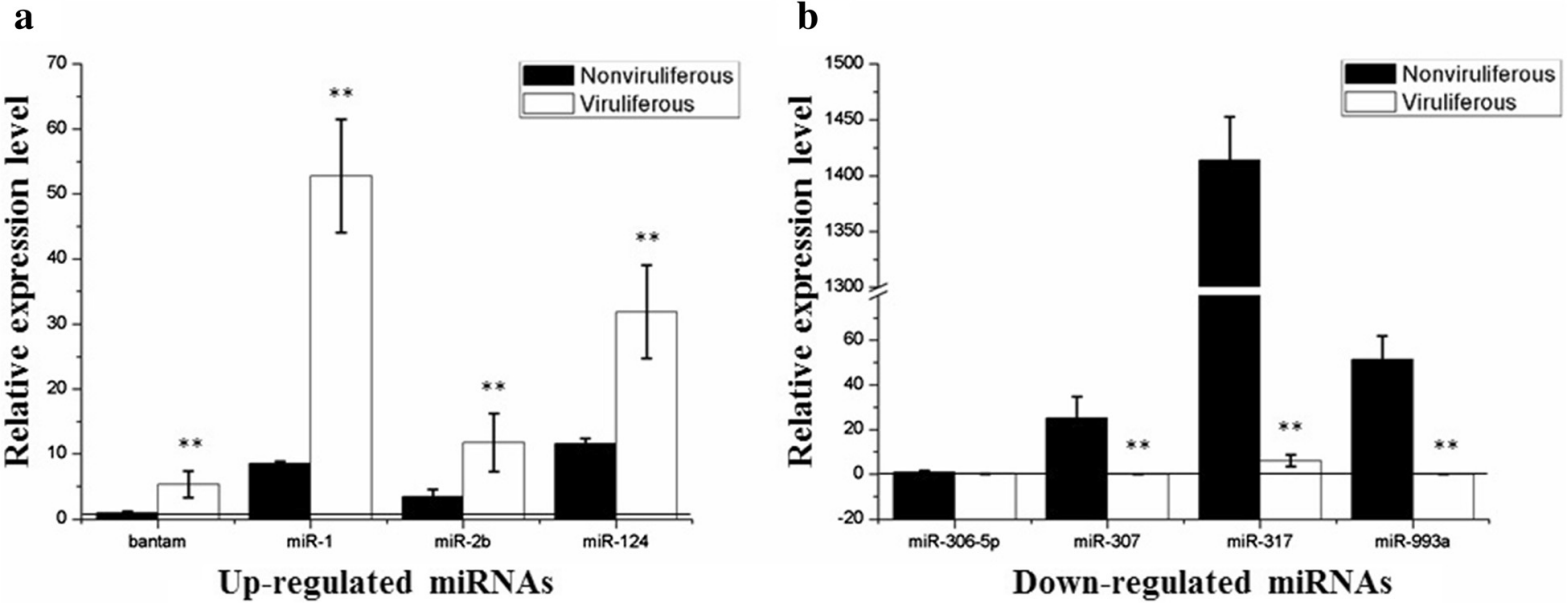
Validation of deregulated conserved miRNA expression levels by quantitative reverse transcription-PCR. The left panel (**a**) shows up-regulated miRNAs and the right panel (**b**) represents down-regulated miRNAs in viruliferous as compared to nonviruliferous whiteflies. The level measured for bantam and miR-306-5p in the nonviruliferous whitefly library was arbitrarily set to 1. Student’s *t*-test in EXCEL was performed and double asterisks indicate a significant difference (*P* < 0.01) between the two-paired samples

To further validate the miRNA sequencing data, we used qRT-PCR to examine the expression levels of four up-regulated miRNAs (bantam, miR-1, −2b, and −124) and four down-regulated miRNAs (miR-306-5p, −307, −317, and −993a) in the viruliferous relative to nonviruliferous library. As shown in Fig. 6, these miRNAs showed an expression pattern consistent with the results obtained from Solexa sequencing (Fig. 4), except miR-306-5p that exhibited similar levels of expression in the two libraries.

**Fig. 7:**
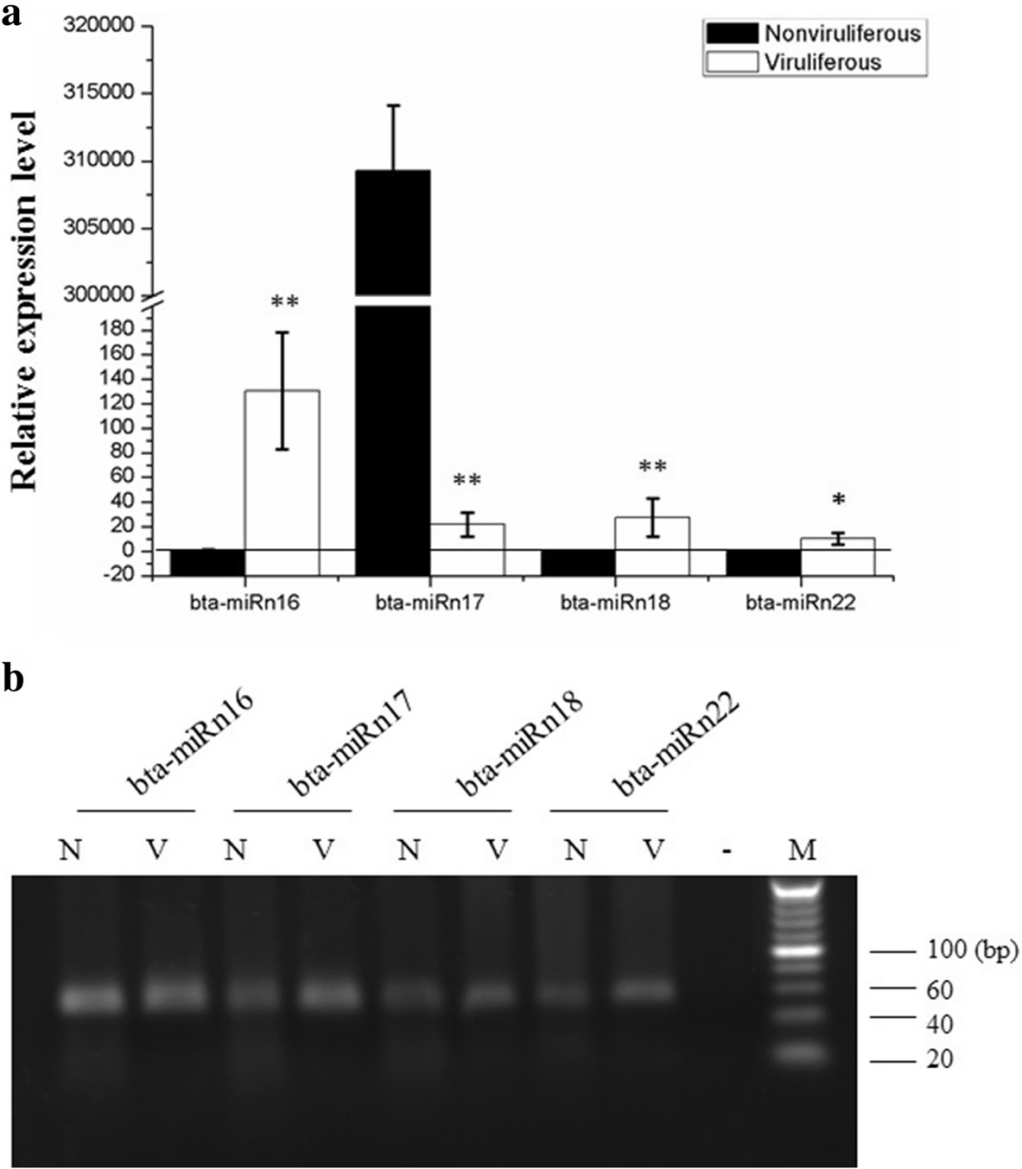
Validation of novel miRNA expression by quantitative reverse transcription-PCR. The diagram (**a**) and the electrophoretogram (**b**) show the novel miRNA comparisons between nonviruliferous and TYLCCNV viruliferous whiteflies. N: nonviruliferous whiteflies, V: TYLCCNV viruliferous whiteflies, M: marker. The level measured for bta-miRn16 in the nonviruliferous whitefly library was arbitrarily set to 1. Student’s *t*-test in EXCEL was performed; single and double asterisks indicate significant difference (*P* < 0.05 and *P* < 0.01, respectively) between the two-paired samples

For the potential novel miRNA candidates identified in both nonviruliferous and viruliferous libraries, we also examined the expression level of four novel miRNAs by qRT-PCR. All of them could be amplified by qRT-PCR using specific primers (Fig. 7). Three of the potential novel miRNA candidates showed higher expression in viruliferous than in nonviruliferous whiteflies, which was the same trend observed in the sequencing data of novel miRNA candidates (Table 1). However, one novel miRNA, bta-miRn17, exhibited a decrease in expression in viruliferous as compared to nonviruliferous whiteflies (Fig. 7a), which is the opposite to that of Solexa sequencing. The reason for these differences is currently unknown.

### miRNA target prediction and GO analysis

The function of a miRNA is ultimately defined by its effects on the expression of target genes. To reveal the possible functions of the miRNAs identified in whiteflies, potential targets of conserved and novel miRNAs were predicted using the previously published *B. tabaci* MEAM1 transcriptome database [46] with the miRNA target prediction algorithm miRanda 3.1. In total 193,090 targets were obtained. The length of the target sequences varied from 200 to 5926 bp, and the binding energy between the miRNAs and the target varied from −20.01 to −48.05 kCal/mol. All of the miRNAs had more than 100 predicted targets. Some miRNAs had more than 1000 predicted targets, and some target genes were putatively regulated by more than two miRNAs. For a better understanding of the functions of the miRNAs, we analyzed the number of miRNA targets in a specific GO. Predicted targets covered three main categories: biological process, cellular component, and molecular function (Additional file 3: Figure S1).

**Fig. 8:**
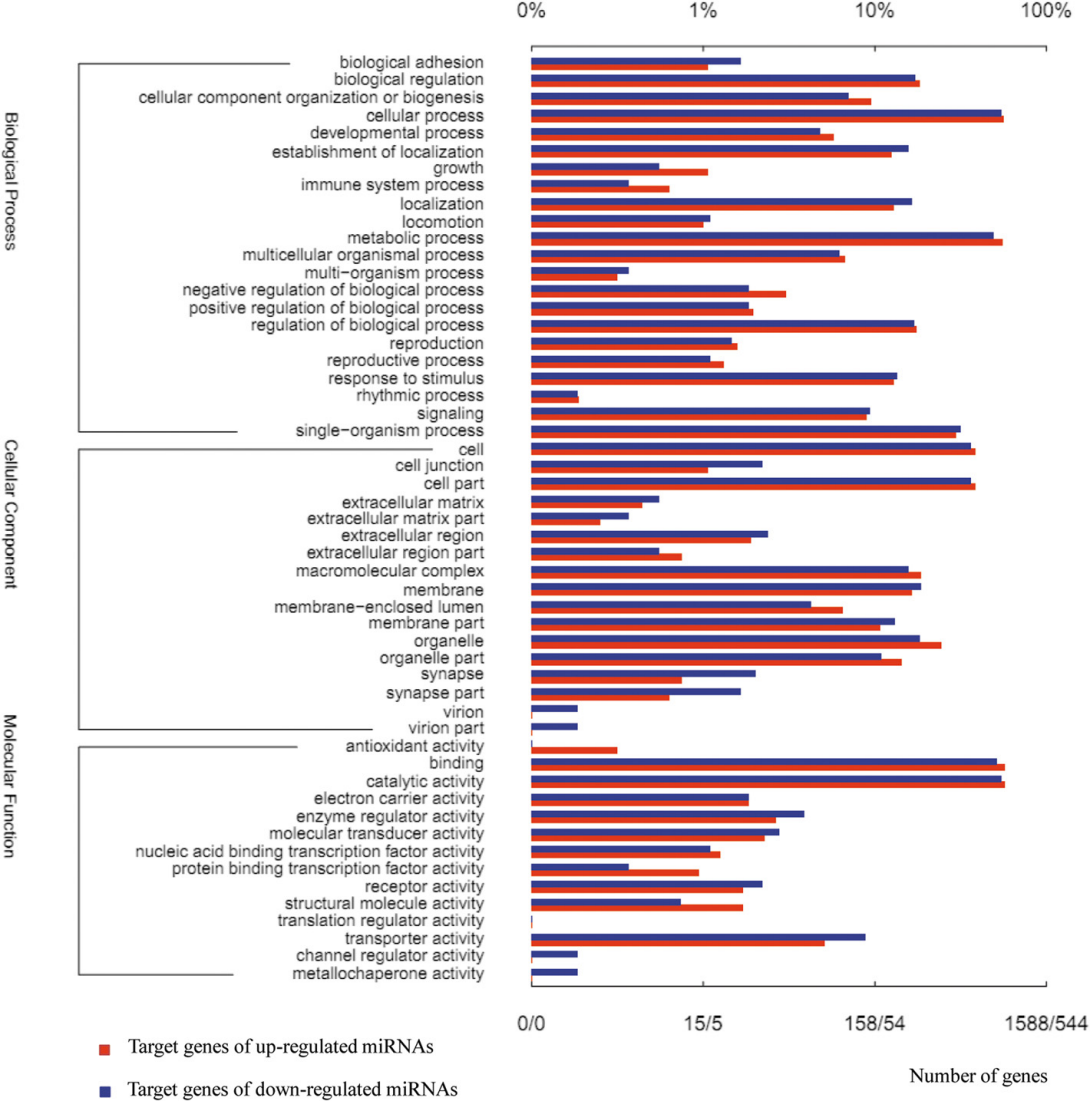
GO classification of putative functions of targets of differentially expressed (up- and down-regulated) miRNAs from the nonviruliferous and viruliferous whitefly libraries. The x axis shows subgroups of molecular functions from GO classification and the y axis shows the number and the percent of the matched unigene sequences

To gain insight into the potential functions of the differentially expressed miRNAs of the nonviruliferous and viruliferous libraries, GO analysis was also used to classify the potential enriched functional groups of their putative targets. For both up- and down-regulated miRNAs in the target libraries, the biological processes most represented are cellular and metabolic processes, whereas binding and catalytic activity are among the most represented molecular function categories, which is similar to the GO analysis of all conserved and novel miRNA targets. To identify the relatively enriched GO terms for up- and down-regulated miRNAs, we first normalized the up- and down-regulated miRNA target genes (normalized target genes = up- or down-regulated miRNA target gene count/total up- or down-regulated miRNA target gene count), and then set a threshold of a 1.5-fold difference in normalized target genes (Table 2). The target genes of up-regulated miRNAs were significantly enriched in eight GO terms, including antioxidant activity, translation regulator activity, protein binding transcription factor activity, structural molecule activity, growth, immune system process, negative regulation of biological process and membrane-enclosed lumen. The targets of down-regulated miRNAs were enriched in nine GO terms, including channel regulator activity, metallochaperone activity, virion, virion part, synapse, synapse part, cell junction, transporter activity and biological adhesion (Fig. 8). Of particular interest is the enrichment of the targets of up-regulated miRNAs in growth. This may be linked to the global transcriptional depression of growth-related genes and contribute to the reduced fecundity and longevity in viruliferous whiteflies [37, 51]. For the targets of novel miRNAs, as compared with the targets of conserved miRNAs, there was a very similar GO distribution (Additional file 4: Figure S2). This suggests that the novel miRNAs might be functionally divergent.

**Table 2:** Go terms of up-regulated and down-regulated miRNA target genes

GO term	Up-regulated miRNA target genes	Down-regulated miRNA target genes	Normalized up-regulated miRNA target genes	Normalized down-regulated miRNA target genes
Biological adhesion	17	9	1.07 %	1.65 %
Biological regulation	291	94	18.32 %	17.28 %
Cellular component organization or biogenesis	152	38	9.57 %	6.99 %
Cellular process	899	295	56.61 %	54.23 %
Developmental process	91	26	5.73 %	4.78 %
Establishment of localization	198	85	12.47 %	15.63 %
Growth	17	3	1.07 %	0.55 %
Immune system process	10	2	0.63 %	0.37 %
Localization	205	89	12.91 %	16.36 %
Locomotion	16	6	1.01 %	1.10 %
Metabolic process	875	266	55.10 %	48.90 %
Multicellular organismal process	106	34	6.68 %	6.25 %
Multi-organism process	5	2	0.31 %	0.37 %
Negative regulation of biological process	48	10	3.02 %	1.84 %
Positive regulation of biological process	31	10	1.95 %	1.84 %
Regulation of biological process	277	92	17.44 %	16.91 %
Reproduction	25	8	1.57 %	1.47 %
Reproductive process	21	6	1.32 %	1.10 %
Response to stimulus	205	73	12.91 %	13.42 %
Rhythmic process	3	1	0.19 %	0.18 %
Signaling	143	51	9.01 %	9.38 %
Single-organism process	471	171	29.66 %	31.43 %
Cell	615	198	38.73 %	36.40 %
Cell junction	17	12	1.07 %	2.21 %
Cell part	615	198	38.73 %	36.40 %
Extracellular matrix	7	3	0.44 %	0.55 %
Extracellular matrix part	4	2	0.25 %	0.37 %
Extracellular region	30	13	1.89 %	2.39 %
Extracellular region part	12	3	0.76 %	0.55 %
Macromolecular complex	294	85	18.51 %	15.63 %
Membrane	260	101	16.37 %	18.57 %
Membrane-enclosed lumen	104	23	6.55 %	4.23 %
Membrane part	171	71	10.77 %	13.05 %
Organelle	387	100	24.37 %	18.38 %
Organelle part	229	59	14.42 %	10.85 %
Synapse	12	11	0.76 %	2.02 %
Synapse part	10	9	0.63 %	1.65 %
Virion	1	1	0.06 %	0.18 %
Virion part	1	1	0.06 %	0.18 %
Antioxidant activity	5	0	0.31 %	0
Binding	906	280	57.05 %	51.47 %
Catalytic activity	905	295	56.99 %	54.23 %
Electron carrier activity	29	10	1.83 %	1.84 %
Enzyme regulator activity	42	21	2.64 %	3.86 %
Molecular transducer activity	36	15	2.27 %	2.76 %
Nucleic acid binding transcription factor activity	20	6	1.26 %	1.10 %
Protein binding transcription factor activity	15	2	0.94 %	0.37 %
Receptor activity	27	12	1.70 %	2.21 %
Structural molecule activity	27	4	1.70 %	0.74 %
Translation regulator activity	1	0	0.06 %	0
Transporter activity	81	48	5.10 %	8.82 %
Channel regulator activity	0	1	0	0.18 %
Metallochaperone activity	0	1	0	0.18 %

## Conclusions

In summary, we identified Ago1 and Dcr1 orthologs from whiteflies, which indicated that miRNA-mediated silencing is present in whiteflies. Our comparative analysis of miRNAs from TYLCCNV viruliferous and nonviruliferous whitefly libraries revealed the relevance of deregulated miRNAs for the post-transcriptional gene regulation in these whiteflies. The potential targets of all expressed miRNAs were predicted. These results will help to acquire a better understanding of the molecular mechanism underlying the complex interactions between begomoviruses and whiteflies.

## Methods

### Whitefly, plant and virus

A colony of the whitefly *B. tabaci* MEAM1 (GenBank accession no. GQ332577) was maintained on cotton (*Gossypium hirsutum* cv. Zhe-Mian 1793) [37] in a climate-controlled chamber at 27 ± 1 °C, with a photoperiod of 14 h light/10 h darkness and relative humidity of 70 ± 10 %. The purity of the colony was monitored every 3–5 generations using the random amplified polymorphic DNA PCR technique combined with sequencing of the *mitochondrial cytochrome oxidase 1* gene, which has been widely used to differentiate species of the whitefly complex [52, 53].

To obtain virus-infected tomato (*Solanum lycopersicum* cv. Hongbaoshi), plants at the 6–8 leaf stage were agroinoculated with an infectious clone of TYLCCNV isolate Y10 (pBinPLUS-Y10 1.7A) in combination with its associated betasatellite (pBinPLUS-2β) at a 1:1 ratio as described previously [32]. Inoculated plants were kept in an insect-free chamber for 21 days post inoculation and then used for the experiments. Infection of test plants was assessed by the appearance of symptoms typical of TYLCCNV and further confirmed by PCR using a procedure described previously [54].

### Identification and cloning of Argonaute 1 and Dicer 1 orthologs from whiteflies

The *Drosophila melanogaster Ago1* and *Dcr1* sequences (GenBank accession no. 36544 and no. 42693) were used to identify whitefly orthologs using the *B. tabaci* transcriptome database [46]. *B. tabaci* MEAM1 cDNAs were generated by reverse transcription using an Oligo (dT) primer, and partial fragments of the *Ago1* and *Dcr1* genes amplified with gene specific primers (Additional file 5: Table S3). The resulting fragments of 2229 and 1440 nucleotides for *Ago1* and *Dcr1*, were then cloned into the pGEM-T Easy vector (Promega, Madison, USA), respectively.

### Sample preparation and RNA extraction

Ten TYLCCNV-infected and ten uninfected tomato plants were placed in insect-proof cages as the inoculum sources [30]. Approximately 10,000 newly emerged adult whiteflies (0–24 h after emergence) were collected from the colony maintained on cotton and released onto either the TYLCCNV-infected tomato plants, or uninfected tomato plants in separate insect-proof cages. The whiteflies were reared under the conditions of temperature, photoperiod and humidity as stated above. Previous studies have demonstrated that MEAM1 whiteflies acquire TYLCCNV rapidly, usually within 12 h of feeding on virus-infected plants [55, 56]. Whiteflies were therefore given an acquisition access period (AAP) of 24 h on both TYLCCNV-infected and uninfected tomato plants, and then transferred to cotton, a non-host plant of TYLCCNV, and reared for 5 days. This procedure was intended to clear as much as possible effects of the test plant on miRNA expression prior to collection for RNA preparation. Whiteflies were tested to verify their status of virus acquisition.

Total RNA was extracted from viruliferous and nonviruliferous whiteflies using TRizol Reagent as described (Invitrogen, Carlsbad, USA). Low molecular weight (LMW) RNAs were enriched using PEG (molecular weight 8000) and NaCl as described [57, 58] and were electrophoresed through a 15 % TBE-urea PAGE gel. The region of the gel containing RNA molecules between 18–28 nt in length was excised and used for small RNA library construction.

### Small RNA library preparation and high-throughput sequencing

Small RNA libraries were constructed as described previously [32, 59]. In brief, 18–28 nt small RNAs were sequentially ligated to a 3′ adapter and a 5′ adapter. After each ligation step, small RNAs were purified using 15 % denaturing PAGE as described above. Subsequently, the final purified ligation products were reverse transcribed into cDNA using Superscript III reverse transcriptase (Invitrogen, Carlsbad, USA). First strand cDNA was amplified by PCR using Taq polymerase (Roche, Basel, Switzerland) and DNA amplicons from each library purified and then separately submitted for high-throughput sequencing using the Solexa platform (Illumina, SanDiego, CA).

### Identification of conserved miRNAs

Tags less than 40 nt were first subjected to data cleaning to remove low quality tags and several kinds of contaminants. The distribution of the lengths of the clean tags was summarized. The clean tags were annotated into different categories using the Rfam Version 10.1; and rRNAs, tRNAs, snRNAs, and snoRNAs were filtered out. The remaining small RNA tags were used to search the latest release of miRBase Version 19.0 to identify conserved miRNAs in *B. tabaci* MEAM1. Conserved miRNAs were defined as sequences present in our libraries that were identical or related to (having four or fewer nucleotide substitutions) sequences from *D. melanogaster* or other insects (*A. aegypti*, *A. mellifera*, *T. castaneum* and *B. mori*) as outlined previously [39].

### Identification of novel miRNAs

Although the characteristic hairpin structure of miRNA precursors could be used to predict novel miRNAs, it is very challenging to define novel miRNAs. We used the prediction software miRDeep2 [48] to predict novel miRNAs. As no completed genome sequences for whiteflies are available, 27,288 nucleotide sequences of *B. tabaci* obtained from the NCBI were used as a reference for the prediction of novel miRNAs as described [39]. We explored the secondary structure, the Dicer cleavage site and the minimum folding free energy of any unannotated small RNA tags that could be mapped to the whitefly genome. To be considered as a potential novel miRNA candidate, the predicted sequences should also meet the default parameters according to miRDeep2. To further evaluate the novel miRNA candidates represent true miRNAs, the secondary structures of putative pre-miRNA hairpins were generated using RNAfold [50].

### Quantitative reverse transcription-PCR (qRT-PCR) of miRNAs

qRT-PCR of miRNAs were conducted as described previously [60]. Briefly, 1 μg of RNase-free DNaseI-treated RNA isolated from viruliferous or nonviruliferous whiteflies was polyadenylated using the Poly (A) Tailing Kit (Ambion, Austin, USA) following the manufacturer’s directions. After phenol-chloroform extraction and ethanol precipitation, the RNAs were reverse-transcribed with 200 U SuperScript™ II Reverse Transcriptase (Invitrogen, Carlsbad, USA) and 0.5 μg poly (T) adapter. For each PCR, 1 μL of template cDNA was mixed with 12.5 μL 2 × SYBR Green PCR master mix (Roche, Basel, Switzerland) and 5 pmoL each of the forward and reverse primers in a final volume of 25 μL. Amplification program was performed as follows: 15 s at 95 °C, followed by 15 s at a temperature 5 °C below the primer’s true Tm, and 20 s at 72 °C for 45 cycles. A thermal denaturing step to generate the dissociation curves was included to verify amplification specificity. All reactions were run in triplicate, and the results were analyzed by the 2^-∆∆CT^ method [61, 62]. Student’s *t*-test in EXCEL was used to analyze the qRT-PCR data of miRNA comparisons between nonviruliferous and viruliferous whiteflies. The primers used in this analysis are listed in Additional file 5: Table S3.

### Target prediction and GO analysis

In the absence of a completed genome sequence, we utilized the previously published *B. tabaci* MEAM1 transcriptome database [46] and the miRNA target prediction algorithm miRanda 3.1 (http://www.microrna.org/microrna/getDownloads.do) to predict potential targets for conserved and novel miRNAs. For miRanda, default parameters were used with the following exceptions: the score was set to ≥130 and the free energy was set to ≤ −16 kCal/mol. Predicted targets were further filtered using more stringent criteria in which they had to contain either (1) a match between nucleotides 2–8 of the miRNA with the target sequence or (2) a match between nucleotides 2–7 and 13–16 of the miRNA with the target sequence (G:U base-pairing was tolerated). To reveal functions related to the putative target genes, Gene Ontology (GO) analysis was performed on predicted target gene candidates for conserved and novel miRNAs and differentially expressed miRNAs using three ontologies: molecular function, cellular components and biological process.

## Additional files

Additional file 1: Table S1. Conserved miRNAs from the nonviruliferous and viruliferous whitefly libraries (DOCX 21 kb) Additional file 2: Table S2. Induced miRNAs only in TYLCCNV viruliferous whiteflies (DOCX 14 kb) Additional file 3: Figure S1. GO classification of putative functions of targets of all conserved and novel miRNAs from the nonviruliferous and viruliferous whitefly libraries. The X axis shows subgroups of molecular functions from GO classification and the Y axis shows the number and the percent of the matched unigene sequences. (DOCX 362 kb) Additional file 4: Figure S2. GO classification of putative functions of targets of novel miRNA candidates from the nonviruliferous and viruliferous whitefly libraries. The X axis shows subgroups of molecular functions from GO classification and the Y axis shows the number and the percent of the matched unigene sequences. (DOCX 190 kb) Additional file 5: Table S3. Sequences of primers used in the study (DOCX 14 kb)
