# Supplementary material for: MicroRNA profiling of the whitefly Bemisia tabaci Middle East-Aisa Minor I following the acquisition of Tomato yellow leaf curl China virus
Source: Virol J. 2016 Feb 2;13:20. doi: 10.1186/s12985-016-0469-7 (PMC4736103; doi:10.1186/s12985-016-0469-7)
Supplement: Additional file 1: Table S1. — Conserved miRNAs from the nonviruliferous and viruliferous whitefly libraries (DOCX 21 kb) [file 12985_2016_469_MOESM1_ESM.docx]

**Additional file 1: Table S1**

**Conserved miRNAs from the nonviruliferous and viruliferous whitefly libraries.**

| Name | Reads | | Normalized reads | |
| --- | --- | --- | --- | --- |
|  | viruliferous | nonviruliferous | viruliferous | nonviruliferous |
| bantam | 114941 | 440 | 264038.1 | 6095.09 |
| bantam-3p | 54 | 0 | 124.05 | 0 |
| let-7 | 15 | 2 | 774.14 | 27.7 |
| let-7a-5p | 9462 | 60 | 21804.66 | 831.15 |
| let-7b-5p | 5 | 0 | 11.49 | 0 |
| let-7c-5p | 43 | 0 | 98.78 | 0 |
| let-7d-5p | 10 | 0 | 22.97 | 0 |
| let-7e-5p | 8 | 0 | 18.38 | 0 |
| let-7f-5p | 4 | 0 | 9.19 | 0 |
| let-7g | 6 | 0 | 13.78 | 0 |
| miR-1 | 10905 | 2994 | 49660.05 | 7480.34 |
| miR-1-3p | 228 | 25 | 1247.36 | 360.16 |
| miR-1b-3p | 1 | 0 | 2.3 | 0 |
| miR-1c | 105 | 8 | 241.2 | 110.82 |
| miR-2a-3p | 152 | 72 | 349.17 | 997.38 |
| miR-2b | 62081 | 6665 | 143705.9 | 92506.85 |
| miR-2b-3p | 107 | 34 | 438.76 | 484.84 |
| miR-2b-2-5p | 1 | 0 | 2.3 | 0 |
| miR-7 | 10602 | 2103 | 24405.05 | 29159.47 |
| miR-8 | 126 | 18 | 344.57 | 443.28 |
| miR-8-5p | 9 | 1 | 20.67 | 27.7 |
| miR-8a-3p | 39 | 5 | 89.59 | 69.26 |
| miR-9 | 8 | 5 | 73.51 | 124.67 |
| miR-9b | 7 | 0 | 16.08 | 0 |
| miR-9b-3p | 303 | 26 | 696.04 | 360.16 |
| miR-9c-5p | 4116 | 1025 | 9455.12 | 14198.79 |
| miR-9e-5p | 1 | 1 | 2.3 | 13.85 |
| miR-10 | 5 | 1 | 11.49 | 13.85 |
| miR-10-3p | 740 | 126 | 1895.16 | 1897.79 |
| miR-10a | 5 | 1 | 11.49 | 13.85 |
| miR-10c | 2 | 2 | 4.59 | 27.7 |
| miR-12 | 26 | 2 | 59.73 | 27.7 |
| miR-12-5p | 32 | 2 | 73.51 | 27.7 |
| miR-13-3p | 42 | 3 | 96.48 | 41.56 |
| miR-13a-3p | 38 | 6 | 87.29 | 83.11 |
| miR-14 | 22500 | 1392 | 52793.38 | 19476.58 |
| miR-29a-3p | 8 | 0 | 18.38 | 0 |
| miR-29b | 5480 | 711 | 12588.45 | 9849.11 |
| miR-31 | 7 | 1 | 16.08 | 13.85 |
| Name | Reads | | Normalized reads | |
|  | viruliferous | nonviruliferous | viruliferous | nonviruliferous |
| miR-33-5p | 5 | 0 | 11.49 | 0 |
| miR-34-5p | 82654 | 8530 | 189986.8 | 118258.6 |
| miR-34a | 54 | 1 | 124.05 | 13.85 |
| miR-71 | 2290 | 770 | 5262.8 | 10680.26 |
| miR-71-3p | 3834 | 98 | 9255.27 | 1357.54 |
| miR-71-5p | 38 | 19 | 87.29 | 263.2 |
| miR-72 | 1154 | 235 | 2650.93 | 3269.18 |
| miR-79 | 1 | 1 | 2.3 | 13.85 |
| miR-79-3p | 303 | 26 | 696.04 | 360.16 |
| miR-80 | 0 | 1 | 0 | 13.85 |
| miR-81 | 2794 | 11 | 6418.27 | 152.38 |
| miR-82 | 283 | 1 | 650.1 | 13.85 |
| miR-87 | 1 | 0 | 2.3 | 0 |
| miR-87a | 162 | 16 | 390.52 | 221.64 |
| miR-87b | 76 | 6 | 176.88 | 83.11 |
| miR-92a | 44 | 2 | 101.08 | 27.7 |
| miR-92a-3p | 2082 | 170 | 4784.99 | 2354.92 |
| miR-92b-3p | 37 | 5 | 85 | 69.26 |
| miR-92c | 551 | 61 | 1265.74 | 845 |
| miR-98-5p | 21 | 0 | 48.24 | 0 |
| miR-99a | 33 | 10 | 78.1 | 138.52 |
| miR-99b-5p | 344 | 164 | 790.22 | 2271.81 |
| miR-100 | 10897 | 2999 | 26472.5 | 43690.72 |
| miR-100-5p | 10897 | 2999 | 26472.5 | 43690.72 |
| miR-100b | 43 | 21 | 98.78 | 290.9 |
| miR-124 | 22806 | 1407 | 52924.32 | 19587.4 |
| miR-125-5p | 11523 | 2927 | 27915.12 | 43108.91 |
| miR-133 | 1586 | 33 | 3645.6 | 457.13 |
| miR-133c | 1 | 0 | 2.3 | 0 |
| miR-137-3p | 2345 | 620 | 5398.33 | 8602.39 |
| miR-137b | 9 | 4 | 20.67 | 55.41 |
| miR-182 | 42 | 18 | 96.48 | 249.34 |
| miR-184 | 8684 | 1851 | 20221.92 | 25890.28 |
| miR-184b | 137 | 52 | 314.71 | 720.33 |
| miR-190 | 2098 | 201 | 4824.04 | 2798.2 |
| miR-200a-3p | 1 | 0 | 2.3 | 0 |
| miR-200b | 64 | 8 | 147.02 | 110.82 |
| miR-200c | 115 | 15 | 264.17 | 207.79 |
| miR-206 | 3 | 1 | 6.89 | 13.85 |
| miR-210 | 4 | 7 | 9.19 | 96.97 |
| miR-210-3p | 14 | 12 | 32.16 | 166.23 |
| miR-219 | 1181 | 18 | 2719.84 | 263.2 |
| Name | Reads | | Normalized reads | |
|  | viruliferous | nonviruliferous | viruliferous | nonviruliferous |
| miR-252-5p | 2 | 1 | 4.59 | 13.85 |
| miR-252a | 2 | 1 | 4.59 | 13.85 |
| miR-252b | 1 | 1 | 2.3 | 13.85 |
| miR-263a | 4292 | 862 | 9859.42 | 11940.84 |
| miR-263a-5p | 4362 | 873 | 10047.79 | 12107.07 |
| miR-263b | 9 | 5 | 20.67 | 69.26 |
| miR-275 | 1992 | 177 | 4807.96 | 2590.41 |
| miR-275-3p | 1992 | 177 | 4764.31 | 2590.41 |
| miR-276-3p | 138475 | 30018 | 318212.1 | 415934.5 |
| miR-276-5p | 3321 | 224 | 7746.03 | 3241.48 |
| miR-276b | 455 | 147 | 1045.21 | 2036.31 |
| miR-277 | 124711 | 16492 | 286481.4 | 228455.1 |
| miR-277-3p | 124710 | 16492 | 286479.1 | 228455.1 |
| miR-278 | 1 | 2 | 2.3 | 27.7 |
| miR-279 | 2 | 1 | 4.59 | 13.85 |
| miR-279b | 1 | 0 | 2.3 | 0 |
| miR-279c | 25 | 20 | 57.43 | 277.05 |
| miR-279d | 4 | 2 | 9.19 | 27.7 |
| miR-281 | 868 | 54 | 9852.53 | 2590.41 |
| miR-281-3p | 0 | 1 | 0 | 13.85 |
| miR-281-5p | 1314 | 285 | 3059.82 | 4044.92 |
| miR-283 | 1 | 0 | 2.3 | 0 |
| miR-285 | 4 | 0 | 9.19 | 0 |
| miR-305 | 9681 | 1521 | 22321.53 | 21138.88 |
| miR-306 | 39 | 61 | 89.59 | 845 |
| miR-306-5p | 76 | 134 | 856.84 | 4973.04 |
| miR-307 | 773 | 378 | 1775.71 | 5236.24 |
| miR-315 | 11 | 11 | 25.27 | 152.38 |
| miR-315-5p | 1541 | 158 | 3638.71 | 2202.54 |
| miR-316 | 671 | 6 | 1548.29 | 83.11 |
| miR-317 | 159225 | 41328 | 365880.5 | 762522.9 |
| miR-375 | 8 | 10 | 18.38 | 138.52 |
| miR-375-3p | 10 | 15 | 275.66 | 761.89 |
| miR-451-5p | 1 | 0 | 2.3 | 0 |
| miR-750 | 9545 | 782 | 21926.41 | 10832.64 |
| miR-750-3p | 165 | 17 | 379.03 | 235.49 |
| miR-927 | 1 | 0 | 2.3 | 0 |
| miR-929 | 17 | 0 | 39.05 | 0 |
| miR-929-5p | 875 | 96 | 0 |  |
| miR-932-5p | 1 | 0 | 2.3 | 0 |
| miR-965 | 55 | 0 | 126.34 | 0 |
| miR-971 | 499 | 4 | 1146.28 | 55.41 |
| Name | Reads | | Normalized reads | |
|  | viruliferous | nonviruliferous | viruliferous | nonviruliferous |
| miR-993 | 6246 | 186 | 14545.63 | 2673.53 |
| miR-993-5p | 1 | 2 | 2.3 | 27.7 |
| miR-993a-5p | 991 | 802 | 2276.49 | 11109.69 |
| miR-993b-3p | 12 | 2 | 27.57 | 27.7 |
| miR-993b-5p | 239 | 182 | 549.02 | 2521.15 |
| miR-996 | 16681 | 10588 | 38318.96 | 146670 |
| miR-998 | 1 | 0 | 2.3 | 0 |
| miR-1000 | 5912 | 1677 | 15046.41 | 24685.12 |
| miR-1175-3p | 1673 | 21 | 3843.15 | 290.9 |
| miR-2765 | 2881 | 431 | 6730.69 | 6108.94 |
| miR-2779 | 91 | 55 | 209.04 | 761.89 |
| miR-2796 | 1983 | 237 | 4557.57 | 3283.04 |
| miR-3049-5p | 1 | 0 | 2.3 | 0 |
| miR-iab-4-3p | 49 | 12 | 112.56 | 166.23 |
| miR-iab-4-5p | 5 | 5 | 11.49 | 69.26 |
