# Supplementary material for: MicroRNA profiling of the whitefly Bemisia tabaci Middle East-Aisa Minor I following the acquisition of Tomato yellow leaf curl China virus
Source: Virol J. 2016 Feb 2;13:20. doi: 10.1186/s12985-016-0469-7 (PMC4736103; doi:10.1186/s12985-016-0469-7)
Supplement: Additional file 2: Table S2. — Induced miRNAs only in TYLCCNV viruliferous whiteflies (DOCX 14 kb) [file 12985_2016_469_MOESM2_ESM.docx]

**Additional file 2: Table S2**

**Induced miRNAs only in TYLCCNV viruliferous whiteﬂies.**

| Name | Reads in libraries | | Name | Reads in libraries | |
| --- | --- | --- | --- | --- | --- |
|  | nonviruliferous | viruliferous |  | nonviruliferous | viruliferous |
| bantam-3p | 0 | 54 | miR-98-5p | 0 | 21 |
| let-7b-5p | 0 | 5 | miR-133c | 0 | 1 |
| let-7c-5p | 0 | 43 | miR-200a-3p | 0 | 1 |
| let-7d-5p | 0 | 10 | miR-279b | 0 | 1 |
| let-7e-5p | 0 | 8 | miR-283 | 0 | 1 |
| let-7f-5p | 0 | 4 | miR-285 | 0 | 4 |
| let-7g | 0 | 6 | miR-451-5p | 0 | 1 |
| miR-1b-3p | 0 | 1 | miR-927 | 0 | 1 |
| miR-2b-2-5p | 0 | 1 | miR-929 | 0 | 17 |
| miR-9b | 0 | 7 | miR-932-5p | 0 | 1 |
| miR-29a-3p | 0 | 8 | miR-965 | 0 | 55 |
| miR-33-5p | 0 | 5 | miR-998 | 0 | 1 |
| miR-87 | 0 | 1 | miR-3049-5p | 0 | 1 |
