# Supplementary material for: MicroRNA profiling of the whitefly Bemisia tabaci Middle East-Aisa Minor I following the acquisition of Tomato yellow leaf curl China virus
Source: Virol J. 2016 Feb 2;13:20. doi: 10.1186/s12985-016-0469-7 (PMC4736103; doi:10.1186/s12985-016-0469-7)
Supplement: Additional file 4: Figure S2. — GO classification of putative functions of targets of novel miRNA candidates from the nonviruliferous and viruliferous whitefly libraries. The X axis shows subgroups of molecular functions from GO classification and the Y axis shows the number and the percent of the matched unigene sequences. (DOCX 190 kb) [file 12985_2016_469_MOESM4_ESM.docx]

**Additional file 4: Figure S2**

GO classification of putative functions of targets of novel miRNA candidates from the nonviruliferous and viruliferous whitefly libraries. The X axis shows subgroups of molecular functions from GO classification and the Y axis shows the number and the percent of the matched unigene sequences.
