# Supplementary material for: MicroRNA profiling of the whitefly Bemisia tabaci Middle East-Aisa Minor I following the acquisition of Tomato yellow leaf curl China virus
Source: Virol J. 2016 Feb 2;13:20. doi: 10.1186/s12985-016-0469-7 (PMC4736103; doi:10.1186/s12985-016-0469-7)
Supplement: Additional file 5: Table S3. — Sequences of primers used in the study (DOCX 14 kb) [file 12985_2016_469_MOESM5_ESM.docx]

**Additional file 5: Table S3**

**Sequences of primers used in the study.**

| Primer | Sequence (5’-3’) |
| --- | --- |
| bta dcr1 F | CATGAGGGAAAACTGAGTCA |
| bta dcr1 R | GCAACTGATTCAAAAACATC |
| bta ago1 F | TGGAAAATGATGCTCAATAT |
| bta ago1 R | CTGCAAAGATAGAAGTCAAA |
| bantam | TGAGATCATCGTGAAAGCTG |
| miR-1 | TGGAATGTAAAGAAGTATGG |
| miR-2b | TATCACAGCCAGCTTTGATG |
| miR-124 | TTAAGGCACGCGGTGAATGC |
| miR-306-5p | TCAGGTACTGAGTGACTCT |
| miR-307 | CACAACCTCCTTGAGTGA |
| miR-317 | TGAACACANCTGGTGGTATC |
| mir993a-5p | TACCCTGTAGATCCGGGC |
| bta-miRn16 | GATGGAGGTTTACTGGTTCT |
| bta-miRn17 | CCCTAAATCAGAGATNTTTGACG |
| bta-miRn18 | CTTGGCCATCCTGACACCCCTT |
| bta-miRn22 | TTACGTACTCAAACAACACAAG |
| 5.8sRNA | CTACGCCTGTCTGAGCGTCGCTT |
| Poly(T) adapter | GCGAGCACAGAATTAATACGACTCACTATAGG(T)12VN |
| Reverse primer | GCGAGCACAGAATTAATACGAC |
